# Combination of pregabalin and Amitriptyline in management of chronic idiopathic pain following penile prosthesis implantation: a pilot study

**DOI:** 10.1186/s12610-024-00223-4

**Published:** 2024-04-02

**Authors:** Hassan Shaker, Nouran Omar El Said, Karim Omar ElSaeed

**Affiliations:** 1 Urology Department, Faculty of Medicine, Ain Shams University, Cairo, Egypt; 2 Pharmacy Practice & Clinical Pharmacy, Faculty of Pharmacy, Future University in Egypt, Cairo, Egypt

**Keywords:** Chronic post-surgical pain, Penile prosthesis, Chronic post-penile prosthesis pain, Pregabalin, Amitriptyline, Douleur chronique post-chirurgicale, Prothèse pénienne, Douleur chronique post-implantatoire, prégabaline, amitriptyline

## Abstract

**Background:**

Chronic post-penile prosthesis pain is de novo pain persisting > 2 months post-operatively. This pain is inadequately reported, poorly understood and undermanaged. The purpose of this current pilot study was to improvise a medical approach to alleviate the condition and assess the combination of Pregabalin and Amitriptyline in its management.

**Results:**

The study enrolled 9 patients complaining of idiopathic penile, pelvic, or scrotal pain persisting > 2 months after penile prosthesis implantation. Patients were prescribed pregabalin 75mg/12h (escalated after 1 week to 150mg/12h upon demand) and Amitriptyline 25mg once daily for 3 months. The pain was reassessed after 10, 30 and 100 days. The dose of pregabalin required and the side effects of the medication were noted. Findings revealed a significant decrease in pain duration (*p* = 0.007), frequency (*p* < 0.001), and intensity (*p* < 0.001); in glanular (*p* = 0.008), shaft pain (*p* = 0.046) but not scrotal (*p* = 0.112). Moreover, a significant decrease was found in sharp pain (*p* = 0.003) and pain aggravated by touch (*p* = 0.008) but not aching pain (*p* = 0.277). Additionally, significant improvement was reported in QoL (*p* < 0.001) and dose escalation of pregabalin to 150mg/12h was required in only 1 case (11%).

**Conclusion:**

The combination of pregabalin and amitriptyline is very effective in the management of chronic idiopathic pain following penile prosthesis implantation. However, due to the ambiguity and lack of reporting of the condition, we recommend a multicentric contribution to acknowledge the condition, and weigh its prevalence accurately, whilst evaluating the efficacy of our approach.

This study received ethical approval from Ain Shams University Research Ethics Committee (REC) FWA 000017585, on 04/13/2023 (REC-FMASU@med.asu.edu.eg).

**Trial registration:**

no FMASU R98/2023.

## Résumé

**Contexte:**

La douleur chronique survenant après l’implantation d’une prothèse pénienne est une douleur de novo qui persiste plus de 2 mois après la chirurgie. Cette douleur est mal rapportée, mal comprise et mal gérée. L’objectif de cette étude pilote était d’improviser une approche médicale pour soulager cette douleur, et d’évaluer l’association de la prégabaline et de l’amitriptyline dans sa prise en charge.

**Résultats:**

L’étude a inclut 9 patients se plaignant de douleurs idiopathiques au pénis, au bassin ou au scrotum, persistantes depuis plus de 2 mois après l’implantation d’une prothèse pénienne. Les patients se sont vu prescrire 75 mg/12 h de prégabaline (augmenté après 1 semaine à 150 mg/12 h sur demande) et 25 mg d’amitriptyline une fois par jour, pendant 3 mois. La douleur a été réévaluée après 10, 30 et 100 jours. La dose de prégabaline requise et les effets secondaires du médicament ont été notés. Les résultats ont montré une diminution significative de la durée de la douleur (*p* = 0,007), de sa fréquence (*p* < 0,001) et de son intensité (*p* < 0,001), au niveau du gland (*p *= 0,008), de la verge (*p *= 0,046) mais pas du scrotum (*p *= 0,112). De plus, une diminution significative a été observée en ce qui concerne la douleur vive (*p* = 0,003) et la douleur aggravée par le toucher (*p* = 0,008), mais pas la douleur aigüe (*p* = 0,277). Enfin, une amélioration significative de la qualité de vie (*p *< 0,001) a été rapportée et l’augmentation de la dose de prégabaline à 150 mg/12 h ne s’est avéré nécessaire que dans 1 cas (11 %).

**Conclusion:**

L’association de la prégabaline et de l’amitriptyline est très efficace dans la prise en charge de la douleur chronique idiopathique suite à l’implantation d’une prothèse pénienne. Cependant, en raison de l’ambiguïté et de l’absence de déclaration de la maladie, nous recommandons la mise en place d’une contribution multicentrique pour reconnaître la maladie et évaluer sa prévalence avec précision, tout en évaluant l’efficacité de notre approche.

Cette étude a reçu l’approbation éthique du Comité d’éthique de la recherche (CER) de l’Université Ain Shams 44 FWA 000017585, le 13/04/2023 (REC46 FMASU@med.asu.edu.eg).

## Background

The definition of chronic pain post penile prosthesis implantation is pain persisting for more than 2 months post-operatively [1]. This pain mimics chronic post-surgical pain (CPSP), where there is no identifiable cause (e.g., malposition, infection); in other words, de novo pain, and is therefore diagnosed by exclusion [2]. This de novo pain is an uncommon and poorly understood topic [3].

Previous studies recorded improvement in chronic pain caused by malposition of the device, particularly cylinder buckling or reservoir migration [4, 5]. On the contrary, de novo chronic pain after penile prosthesis implantation was irresponsive to surgical revision [6], and therefore we aimed to improvise medical approaches to alleviate the condition.

We chose the combination of pregabalin and amitriptyline as it has been favored by some urologists in tackling resistant cases of Chronic Pelvic Pain Syndrome (CPPS). Its efficacy was also studied previously as part of a multi-modal approach to post-penile prosthesis pain, yet without any differentiation as to whether the pain was de novo or due to a correctable cause [7–9]. Our pilot study aimed to shed light on the condition and test the efficacy and safety of the aforementioned combination.

### Subjects and methods

This pilot study was carried out in Ain Shams University Hospitals and all work was conducted with the approval of our institutional ethics committee; Trial Registration No.: FMASU R98/2023*.* Patients enrolled in our study complained of new penile, pelvic, or scrotal discomfort persisting over two months after penile prosthesis implantation. Patients who required re-operation due to erosion or infection were excluded, as well as cases with malposition or signs of improper size. Our targets were idiopathic subjects only.

History taking: Detailed history with specific attention to age, Diabetes Mellitus (DM), Body Mass Index (BMI), and symptoms of CPPS preoperatively.

Any intraoperative findings were considered, especially excessive corporal scarring.

Pain was analyzed: duration of pain episodes, frequency of pain episodes, location of pain, character of pain, pain aggravated by touch, pain intensity by Visual Analog Scale (VAS) [10], impact on Quality Of Life (QOL), any prior pain killers and their response (opioids, NSAIDs). To avoid using largely subjective and rather complex questionnaires, nevertheless not disease-specific, QOL was simply expressed as satisfactory or unsatisfactory [11].

After signing a written consent, patients were prescribed pregabalin 75mg twice daily (to be escalated after 1 week to 150mg twice daily upon demand) and Amitriptyline 25mg once daily, for a 3-month course.

From initiation of medication, the pain was reassessed again after 10, 30 and 100 days (10 days after cessation of medication), as follows: duration of pain episodes, frequency of pain episodes, location of pain, character of pain, if pain was aggravated by touch, pain intensity by VAS, impact on QOL, dose of pregabalin required to relieve pain, side effects of medication.

## Statistical methods

Statistical analysis and graphing were performed using Microsoft Excel version 2016 and SPSS for Windows version 24. Data were described as range, mean ± standard deviation for continuous variables; range, median and interquartile range for numeric discrete variables; or frequency and percentage for categorical variables. Overall statistical differences between related measurements immediately postoperatively, and at 10 days, 30 days and 100 days postoperatively were calculated using Cochran’s Q-test (for categorical variables); or Friedman’s two-way test (for numeric variables). Pair-wise analysis was further performed for measurements that showed statistically significant differences in the overall analysis. The significance level was set at 0.05; yet adjusted *p*-values were expressed to avoid the error of multiple testing in pair-wise comparisons.

## Results

Before any data interpretation, let us present a common complaint that might shed light on such a group of patients so that we can identify them more easily:

‘Glans penis sharp pain mimicking an electric taser, scrotal pain, penile base pain but on the sides near the cylinders, penile pain extremely sensitive to touch, especially ventral surface, baseline pain extremely uncomfortable, agonizing at frequent instances, with remarkable impact on QOL.’ These were quoted directly from patients’ complaints.

**Fig. 1:**
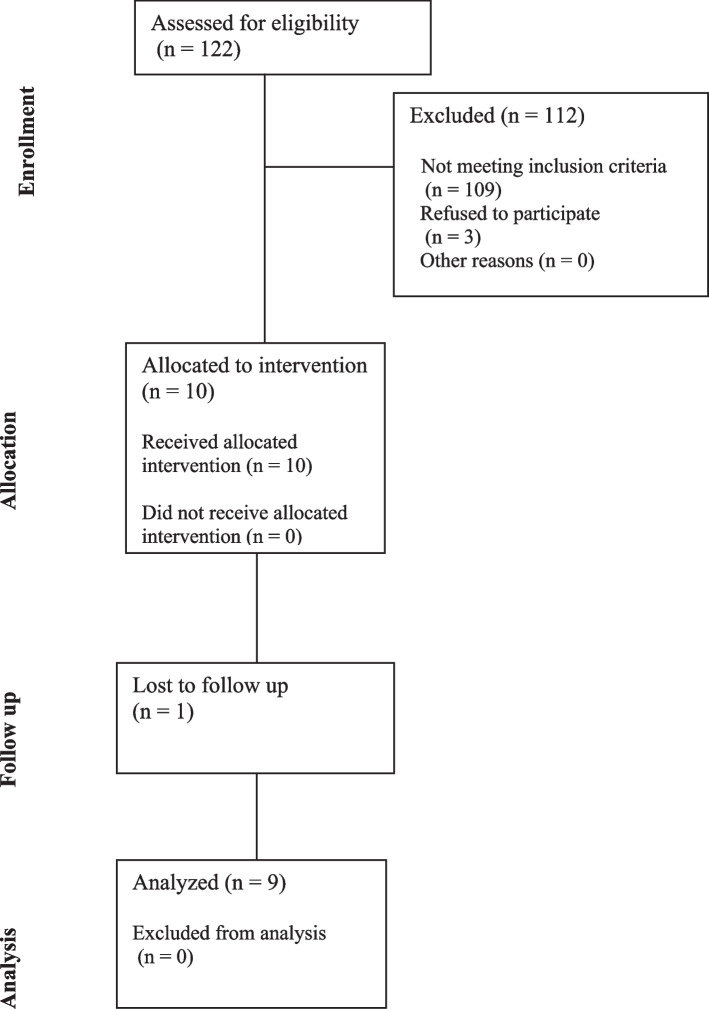
Study diagram showing participant recruitment

A total of 122 cases were performed in our center in the last 6 months, 103 applied the semirigid penile prosthesis and 19 applied the 3-piece inflatable penile prosthesis. The inclusion criteria enrolled 9 cases in this study (10 in total, with 1 lost during follow-up). The study flow diagram for recruitment is shown in Fig. 1.

Table 1 shows the baseline characteristics of included cases, the type of prosthesis used and the incidence of corporal scarring. Table 2 shows the immediate postoperative pain outcomes in the included cases.

**Table 1:** Baseline characteristics of included cases

Age (years)	
Range	48 – 71
Mean ± SD	59.56 ± 8.01
BMI (kg/m^2^)	
Range	23 – 32
Mean ± SD	28.22 – 2.86
Diabetes Mellitus	
Yes	4 (44.4%)
No	5 (55.6%)
Preoperative CPPS	
1	1 (11.1%)
0	8 (88.9%)
Previous Analgesia	
NSAIDs	7 (77.8%)
Opioid	2 (22.2%)
Type of Prosthesis	
Semi-rigid	7 (77.8%)
Inflatable	2 (22.2%)
Corporal Scarring	
Yes	1 (11.1%)
No	8 (88.9%)

*SD* standard deviation, *BMI* body mass index, *CPPS* chronic pelvic pain score, *NSAIDs* non-steroidal anti-inflammatory drugs

Data presented as range, mean ± SD; or frequency (percentage)

**Table 2:** Immediate postoperative pain outcomes in included cases

Duration of Pain Episode (min)	
Range	15 – 60
Mean ± SD	30.0 ± 12.99
Frequency of Pain Episodes	
Range	2 – 7
Median (IQR)	4 (3 – 5)
Location of Pain	
Glans	8 (88.9%)
Shaft	6 (66.7%)
Scrotum	2 (22.2%)
Character of Pain	
Sharp	7 (77.8%)
Aching	2 (22.2%)
Pain Aggravated by Touch	
Yes	7 (77.8%)
No	2 (22.2%)
VAS for Pain	
Range	4 – 7
Median (IQR)	5 (5 – 7)
QoL	
Satisfied	1 (11.1%)
Unsatisfied	8 (88.9%)

S*D* standard deviation, *IQR* interquartile range, *VAS* visual analogue scale, *QoL* quality of Life

Data presented as range, mean ± SD; range, median (IQR); or frequency (percentage)

Of the included 9 cases, 8 (88.9%) received pregabalin at a dose of 150 mg per day, while 1 (11.1%) received it at a dose of 300 mg per day; 8 (88.9%) had drowsiness 10 days postoperatively, while 7 (77.8%) had drowsiness 30 days postoperatively.

Regarding, the duration of pain episodes ≥ 15 min, the difference was statistically significant in the overall analysis (*p* = 0.007). Pair-wise comparisons showed that statistically significant differences existed in comparisons between immediate and at 10 days postoperatively (*p* = 0.028); immediate and at 30 days postoperatively (*p* = 0.028); and immediate and at 100 days postoperatively (*p* = 0.028) (Tables 3 and 4).

**Table 3:** Difference between pain outcomes [immediate, 10 days, 30 days and 100 days postoperatively] in included cases

	**Immediate** **Postop**	**10 Days** **Postop**	**30 Days** **Postop**	**100 Days** **Postop**	***P***
Duration of Pain Episode (min)					
None	0 (0%)	3 (33.3%)	3 (33.3%)	4 (44.4%)	0.007^a^ S
< 15 min	0 (0%)	5 (55.6%)	5 (55.6%)	4 (44.4%)	
≥ 15 min	9 (100%)	1 (11.1%)	1 (11.1%)	1 (11.1%)	
Frequency of Pain Episodes					
Range	2 – 7	0 – 5	0 – 4	0 – 4	< 0.001^b^ S
Median (IQR)	4 (3 – 5)	1 (0 – 3)	1 (0 – 3)	1 (0 – 2)	
Location of Pain					
Glans	8 (88.9%)	4 (44.4%)	4 (44.4%)	3 (33.3%)	0.008 (S)^a^
Shaft	6 (66.7%)	4 (44.4%)	4 (44.4%)	2 (22.2%)	0.046 (S)^a^
Scrotum	2 (22.2%)	0 (0%)	0 (0%)	0 (0%)	0.112 (NS)^a^
Character of Pain					
Sharp	7 (77.8%)	1 (11.1%)	4 (44.4%)	1 (11.1%)	0.003 (S)^a^
Aching	2 (22.2%)	5 (55.6%)	2 (22.2%)	4 (44.4%)	0.277 (NS)^a^
Pain Aggravated by Touch					
Yes	7 (77.8%)	3 (33.3%)	3 (33.3%)	2 (22.2%)	0.008^a^ S
No	2 (22.2%)	6 (66.7%)	6 (66.7%)	7 (77.8%)	
VAS for Pain					
Range	4 – 7	0 – 3	0 – 2	0 – 1	< 0.001^b^ S
Median (IQR)	5 (5 – 7)	1 (0 – 2)	1 (0 – 1)	1 (0 – 1)	
QoL					
Satisfied	1 (11.1%)	8 (88.9%)	9 (100%)	9 (100%)	< 0.001^a^ S
Unsatisfied	8 (88.9%)	1 (11.1%	0 (0%)	0 (0%)	

*SD* standard deviation, *IQR* interquartile range, *VAS* visual analogue scale, *QoL* quality of Life, *S* significant, *NS* non-significant

Data presented as range, mean ± SD; range, median (IQR); or frequency (percentage)

^a^Analysis using Cochran’s Q-Test

^b^Analysis using Friedman’s Two-Way Test

**Table 4:** Pair-wise differences between pain outcomes [immediate, 10 days, 30 days and 100 days postoperatively] in included cases

	**Immediate vs** **10 Days Postop**	**Immediate vs** **30 Days Postop**	**Immediate vs** **100 Days Postop**	**10 Days vs** **30 Days Postop**	**10 Days vs** **100 Days Postop**	**30 Days vs** **100 Days Postop**
Pain Episode Duration ≥ 15 min	0.028 S	0.028 S	0.028 S	1.000 NS	1.000 NS	1.000 NS
Frequency of Pain Episodes	0.082 NS	0.037 S	0.001 S	1.000 NS	0.865 NS	1.000 NS
Location of Pain						
Glans	0.068 (NS)	0.068 (NS)	0.009 (S)	1.000 (NS)	1.000 (NS)	1.000 (NS)
Shaft	0.944 (NS)	0.944 (NS)	0.028 (S)	1.000 (NS)	0.944 (NS)	0.944 (NS)
Character of Pain Sharp	0.008 (S)	0.653 (NS)	0.008 (S)	0.653 (NS)	1.000 (NS)	0.653 (NS)
Pain Aggravated by Touch	0.068 NS	0.068 NS	0.009 S	1.000 NS	1.000 NS	1.000 NS
VAS for Pain	0.028 S	0.006 S	0.001 S	1.000 NS	1.000 NS	1.000 NS
Satisfied QoL	0.004 S	0.001 S	0.001 S	1.000 NS	1.000 NS	1.000 NS

*VAS* visual analogue scale, *QoL* quality of Life, *S* significant, *NS* non-significant

Analysis using Post-Hoc Pair-Wise Analysis (adjusted *p* values are presented)

Nevertheless, regarding the frequency of pain episodes, the difference was statistically significant in the overall analysis (*p* < 0.001). Pair-wise comparisons showed that statistically significant differences existed only in comparisons between immediate and 30-day postoperatively (*p* = 0.037); and immediate and 100-day postoperatively (*p* = 0.001) (Tables 3 and 4).

Whilst comparing the location of pain at the glans, there was a statistically significant difference in overall analysis (*p* = 0.008). Pair-wise comparisons showed that statistically significant differences existed only in comparison between immediate and 100-day postoperatively (*p* = 0.009) (Tables 3 and 4).

Regarding the location of pain at the shaft, the difference was statistically significant in the overall analysis (*p* = 0.046). Pair-wise comparisons showed that statistically significant differences existed only in comparison between immediate and 100-day postoperatively (*p* = 0.028) (Tables 3 and 4).

On the other hand, comparing the location of pain at the scrotum, the difference was statistically insignificant in the overall analysis (*p* = 0.112) (Table 3).

As for the frequency of sharp pain, the difference was statistically significant in the overall analysis (*p* = 0.003). Pair-wise comparisons showed that statistically significant differences existed only in comparisons between immediate and 10-day postoperatively (*p* = 0.008); and immediate and at 100 days postoperatively (*p* = 0.008) (Tables 3 and 4).

On the contrary, the frequency of aching pain showed a statistically insignificant difference in overall analysis (*p* = 0.277) (Table 3).

As regards the frequency of pain aggravated by touch, the difference was statistically significant in the overall analysis (*p* = 0.008). Pair-wise comparisons showed that statistically significant differences existed only in comparison between immediate and 100-day postoperatively (*p* = 0.009) (Tables 3 and 4).

In the meantime, comparing VAS for pain showed a statistically significant difference in overall analysis (*p* < 0.001). Pair-wise comparisons showed that statistically significant differences existed only in comparisons between immediate and at 10 days postoperatively (*p* = 0.028); immediate and at 30 days postoperatively (*p* = 0.006); and immediate and at 100 days postoperatively (*p* = 0.001) (Tables 3 and 4).

Last but not least, the QoL satisfaction comparison showed a statistically significant difference in overall analysis (*p* < 0.001). Pair-wise comparisons showed that statistically significant differences existed only in comparisons between immediate and at 10 days postoperatively (*p* = 0.004); immediate and at 30 days postoperatively (*p* = 0.001); and immediate and at 100 days postoperatively (*p* = 0.001) (Tables 3 and 4).

## Discussion

Despite penile prosthesis being acceptable to patients with satisfactory long-term outcomes, following the implantation, de novo chronic pain may be experienced post-operatively that does not respond to surgical revision [6, 12].

The main predicament is that the proportion of men who will develop chronic pain after prosthesis implantation is completely unknown, with very scanty comparable published data and an extreme lack of reporting cases with prolonged post-prosthesis pain and their surgical intervention if any [13].

Our pilot study aimed primarily to acknowledge the condition and test the efficacy and safety of combining pregabalin and amitriptyline in the management of chronic idiopathic pain following penile prosthesis implantation. Our results were very promising and showed decreased pain episodes as regards duration, frequency, and intensity (as demonstrated by VAS). This was seen mainly with glanular and shaft pain but not scrotal; in sharp pain as well as pain aggravated by touch but not aching pain. There was also a statistically significant improvement in the quality of life.

In the same context, in a study by Campbell et al., 31 patients were diagnosed with chronic penile prosthesis pain after at least 2 months from surgery, and after excluding infection and malposition. A total of 18 patients were scheduled for revision and 13 for explantation. The choice of revision versus explantation was influenced primarily by the patient’s preference. Only, 18 patients (58%) showed no postoperative relief of pain, and their surgeries showed no device malposition, while 13 patients (42%) showed a positive response, which was owed to an intraoperative finding of device malposition. Patients who experienced pain relief, initially suffered penile pain (84.6%), whereas those with persistent pain suffered pelvic pain (25%) or scrotal pain (38%). They therefore concluded that penile pain may be an indicator of device malposition. In their study, the only factor showing statistical significance in predicting the development of postoperative chronic penile prosthesis pain is a prior diagnosis of chronic pain. Furthermore, based on their findings they also categorized patients with chronic penile prosthesis pain into anatomic (surgically correctable etiology) or idiopathic (non-identifiable etiology analogous to CPSP). They therefore concluded that no operative intervention should be granted with no evidence of device malposition. Another notable finding worth mentioning is that 5 patients had persistent postoperative pain despite being preoperatively diagnosed with device malposition; 2 of which had a positive history of chronic pain syndrome while 2 others were previously on opioids. Campbell and his colleagues, although helped to shed light on the condition, and advised against any surgical approach to eradicate the condition as they proved it non-beneficial, they however did not provide any solid alternative solutions [6].

The hypothesis generated to support the development of chronic penile prosthesis pain is the flare of an immune/inflammatory reaction triggered by operative violation of nerve axons. This in turn creates a cascade starting with local neurotransmitter release and ending with central sensitization [14]. This pathophysiology mimics the neuropathic pain of CPSP and hence may propose a non-surgical approach to patients with chronic penile prosthesis pain where neither infection nor malposition could be accused. So what alternatives are we left with? Multimodal analgesia including gabapentinoids, tricyclic antidepressants, or serotonin-norepinephrine reuptake inhibitors may be effective and minimize narcotic usage [8, 9, 15, 16]. Other approaches include behavioral and physical therapies (physiotherapy, acupuncture, etc..) [9, 17, 18].

In 2018, Tong and colleagues introduced the multimodal analgesia protocol in the management of pain following inflatable penile prosthesis implantation [8]. A few more studies refined the technique and tested its efficacy [9, 16]. The same approach along with intraoperative nerve block was further investigated and outcomes were reported for a longer period of 6 weeks postoperatively [19] to address the lack of literature for outcome assessment beyond a month [20]. Gabapentinoids were used as part of the multimodal approach, and their results are as enthusiastic as ours, however non-comparable to us for a couple of reasons. Firstly, their approach did not target or acknowledge chronic patients with do novo pain after 2 months postoperatively, neither did they use gabapentin solely to alleviate the pain and therefore give us comparable material [9, 16, 20].

### Limitations

The current study was a pilot trial of a hypothesized management approach, for a poorly reported condition, lacking standard treatment as a benchmark. Due to the ambiguity of the condition, lack of recognition as well as lack of reporting, our evaluation may not represent the entire population. The limitations include its small sample size and non-comparative, single-centred nature. Moreover, the variability in penile prosthesis implant type used by enrolled patients may deter accurate outcome assessment.

## Conclusions

The combination of pregabalin and amitriptyline was found very effective in the management of chronic idiopathic pain following penile prosthesis implantation. The aforementioned combination managed to decrease pain episodes concerning duration, intensity and frequency, with a substantial improvement in the quality of life. Dose upgrades of pregabalin were rarely used, and therefore we can conclude that the optimum starting dose is 75mg/12h. We recommend a multicentric contribution to acknowledge the complication and weigh its prevalence accurately while evaluating the efficacy of our approach to tackling such a burden.

## Data Availability

Data generated or analyzed during this study can be found in the published article. Any additional data is available from the corresponding author upon reasonable request.

## Abbreviations

BMI: Body Mass Index
CPPS: Chronic Pelvic Pain Syndrome
CPSP: Chronic Post-Surgical Pain
DM: Diabetes Mellitus
QOL: Quality Of Life
VAS: Visual Analog Scale
